# Human brain zero: uncovering the lowest neural state for consciousness engineering

**DOI:** 10.3389/fnins.2026.1819370

**Published:** 2026-05-08

**Authors:** Maël Donoso

**Affiliations:** Ouroboros Neurotechnologies, Lausanne, Switzerland

**Keywords:** anesthesia, brain foundation models, consciousness engineering, human brain zero (HB_0_), neural state transitions, neuroimaging, sleep, wakefulness

## 1 Introduction

The novel domain of *consciousness engineering* considers consciousness as an emergent property, sustained by organized processes within a particular class of complex adaptive systems, which could be either biological or artificial. Under this assumption, the relatively continuous stream of experience associated with human consciousness could be described as a trajectory through time, which continues as long as the underlying system, the human brain, remains functional. Following this idea, the termination of this trajectory at the end of a human life could be seen as a biological contingency, rather than a physical necessity. Theoretically, if the processes sustaining human consciousness were to be transferred to a different, potentially more robust physical substrate, all the while preserving the relative continuity of the stream of experience, human cognition might be able to persist beyond biological death. Practically, such an endeavor would almost certainly require significant scientific and technological advances, such as a deeper understanding of the neural foundations of human consciousness, as well as the development of novel methods spanning neuroengineering, materials science, and computer science. While a realistic implementation of such substrate transition is probably better seen as a long-term objective, establishing consciousness continuity as a falsifiable, experimentally testable scientific target offers a unique opportunity to engage in ambitious, interdisciplinary research on this emergent property of the human brain, and to develop new principles and methods to investigate conscious experience.

Consciousness engineering is most certainly a challenging problem—but it does not need to be more challenging than necessary. Theoretical models of human consciousness in neuroscience, such as the global neuronal workspace model (Dehaene and Changeux, 2011), typically associate conscious experience with the engagement of a large-scale functional network involving multiple brain regions, particularly in the prefrontal cortex. Arguably, the degree of complexity and integration of this functional network would represent one of the main difficulties in any serious attempt at substrate transition, regardless of the exact definition of complexity, integration, or consciousness. However, even if conscious states are defined by the activation of a complex and integrative network, humans routinely experience the fact that consciousness can reemerge from a variety of other neural states, such as deep sleep. As these unconscious states are sometimes associated with simpler and more local patterns of brain activity, a fundamental scientific question for consciousness engineering could be the following: what would be the lowest neural state, in terms of complexity and integration, that would still allow for the reemergence of consciousness in a biological brain? For our species, we propose to call this hypothetical neural state: *human brain zero* (HB_0_).

By contrast with scientifically identified neural states, such as wakefulness or sleep, whose characteristics can be investigated directly, HB_0_ is intended to serve as a heuristic construct for orienting future theoretical and experimental research on consciousness engineering. In this sense, HB_0_ should be understood as an ideal neural state, a hypothetical limit whose characteristics can only be uncovered by investigating empirical approximations: HB_0_ candidates. Uncovering HB_0_, a neural state potentially lower than sleep, anesthesia, or reversible coma, could provide a lower bound for the consciousness engineering problem, as any conceivable technology for substrate transition should probably, at the very least, succeed when the functional network associated with consciousness is reduced to its simplest form. Therefore, on the path to solving the consciousness engineering problem, a promising starting point could be to define the conditions under which this problem becomes the easiest, by using currently available neuroimaging technologies to discover or predict HB_0_ candidates, without waiting for the future technologies that would be necessary to implement a successful substrate transition. As HB_0_ is primarily defined by the degree of complexity and integration within the human brain, it is designed not to depend too strongly on any specific theoretical model of consciousness, assuming of course that normal consciousness, to which the human brain is meant to return after reaching HB_0_, can be considered consensual enough to be similarly recognized by every theory. While the precise interpretation of HB_0_ may still depend on theoretical assumptions about human consciousness, the objective of this conceptual design is to keep this heuristic construct as flexible as possible regarding specific theoretical models. By contrast, HB_0_ is directly dependent on the ability to measure or approximate complexity and integration in the human brain, as we will now discuss.

## 2 Neural states

The strategy consisting in uncovering HB_0_ could change the focus of consciousness engineering, from the continuation of organized processes to the preservation of the capacity to restart these processes. Neuroscience research provides important insights into the trajectories that the human brain can follow to return from lower, presumably unconscious neural states to higher, primarily conscious neural states, but before considering these trajectories, the intuitive notions of “low” and “high” complexity and integration must be defined more precisely. As our understanding of the neural foundations of human consciousness remains limited, any attempt to formalize these notions is necessarily speculative, but we can think of several neuroimaging metrics that could serve as proxies for evaluating the degree of complexity and integration of a given neural state. These proxies will allow us to ground the discussion of HB_0_ in existing neuroscience literature, even if further research would be needed to confirm their relevance for consciousness engineering. Focusing on sleep, a first proxy could be reactivity: electrical responses to transcranial magnetic stimulation (TMS) propagate during wakefulness but are rapidly extinguished during sleep (Massimini et al., 2005), and the complexity of these response patterns reliably predicts the level of consciousness (Casali et al., 2013; Nieminen et al., 2016). A second proxy could be hemodynamic activity: functional magnetic resonance imaging (fMRI) studies have associated sleep with altered correlations (Horovitz et al., 2009), reduced temporal complexity (Tagliazucchi et al., 2013), and decreased effective connectivity (Jobst et al., 2017) within the human brain, particularly in a fundamental network for consciousness, the default mode network (DMN). Multimodal research combining positron emission tomography, electroencephalography (EEG), and fMRI has further confirmed the reduction of hemodynamic activity in the DMN (Chen et al., 2025). Finally, a third proxy could be electrical activity: multimodal research combining scalp EEG, intracerebral EEG, and single-unit recording has demonstrated that human brain activity during sleep is predominantly local (Nir et al., 2011).

These proxies may hold for other neural states as well. Studies on anesthesia have reported lower reactivity to TMS (Ferrarelli et al., 2010), decreased functional connectivity (Boveroux et al., 2010), and reduced neuronal activity within several brain regions (Krom et al., 2020). Studies on patients in a minimally conscious state (Schiff et al., 2007), where consciousness is partially preserved, or a vegetative state (Boly et al., 2011), characterized by unresponsive wakefulness, have both highlighted the importance of connectivity, as well as the prominent role of the frontal cortex in normal consciousness. The objective of uncovering HB_0_ may also benefit from the exploration of other neural states of the human brain, such as the altered conscious states temporarily induced by meditation (Brewer et al., 2011), hypnosis (McGeown et al., 2009), and the effects of psychedelic drugs (Carhart-Harris et al., 2012, 2016), which have all been associated with reduced activity or integration in the DMN. Sleep itself can be decomposed into different stages, and encompasses a diversity of particular neural states, such as dreaming (Siclari et al., 2017; Bréchet et al., 2020) or lucid dreaming (Dresler et al., 2012), correlated with specific neural patterns in the human brain. Finally, the definition of a trajectory toward HB_0_ may benefit from the exploration of neural states that do not naturally occur in our species, such as hibernation. In particular, research on hibernating primates has been highlighted as a promising path for the hypothetical induction of hibernation-like states in humans (Blanco et al., 2016).

Theoretically, based on these proxies, HB_0_ could be either *discovered* if the lowest neural state can be directly measured, or *predicted* if it can only be inferred. Practically, as HB_0_ serves as a lower bound, it represents in fact a moving scientific target: the objective should not be to prove the existence of this neural state, but rather to identify, at any given time, which discovered or predicted neural state is the closest empirical approximation to HB_0_, making it possibly the best option for consciousness engineering. The existence of HB_0_ may not be falsifiable, but identifying a particular neural state as HB_0_ is: falsifiability of HB_0_ candidates would only require the discovery or prediction of another neural state with lower complexity or integration metrics. The proxies selected for approximating complexity and integration could be debated, and the precise interpretation of HB_0_ may depend on broader assumptions about human consciousness, but the idea of an ordering of neural states based on neuroimaging metrics could provide, in any case, a useful and adaptable framework for the consciousness engineering problem.

## 3 Consciousness transitions

Let us assume that neuroimaging metrics such as reactivity, hemodynamic activity, and electrical activity could serve as useful proxies for characterizing lower and higher neural states in terms of complexity and integration. The next step toward the discovery or prediction of HB_0_ candidates would likely be to deepen our understanding of the possible trajectories between those neural states, and if possible, to map consciousness transitions to specific changes in complexity or integration throughout the human brain. Of particular relevance here, research using simultaneous cortical and subcortical recording (Magnin et al., 2010) and multimodal research combining EEG and fMRI (Stevner et al., 2019; Song et al., 2022) have characterized the trajectory from wakefulness to sleep, which typically starts with functional alterations in the thalamus before extending to the posterior cortex and frontal cortex. Reciprocally, fMRI studies (Setzer et al., 2022), EEG studies (Stephan et al., 2025), and multimodal studies combining EEG and fMRI (Song et al., 2022) have characterized the reverse trajectory from sleep to wakefulness, which also typically starts with the thalamus and is associated with the progression of specific functional patterns throughout the cortex. Importantly, these consciousness transitions are not immediate: the progressive loss of responsiveness while falling asleep has been associated with distinctive electrical responses in EEG and magnetoencephalography (Strauss et al., 2022), while the sleep inertia experienced after awakening has been similarly correlated with specific functional patterns in EEG and fMRI (Vallat et al., 2019; Wang et al., 2024). Together, these discoveries suggest that the transitions between neural states may follow, to some extent, predictable trajectories.

**Figure 1:**
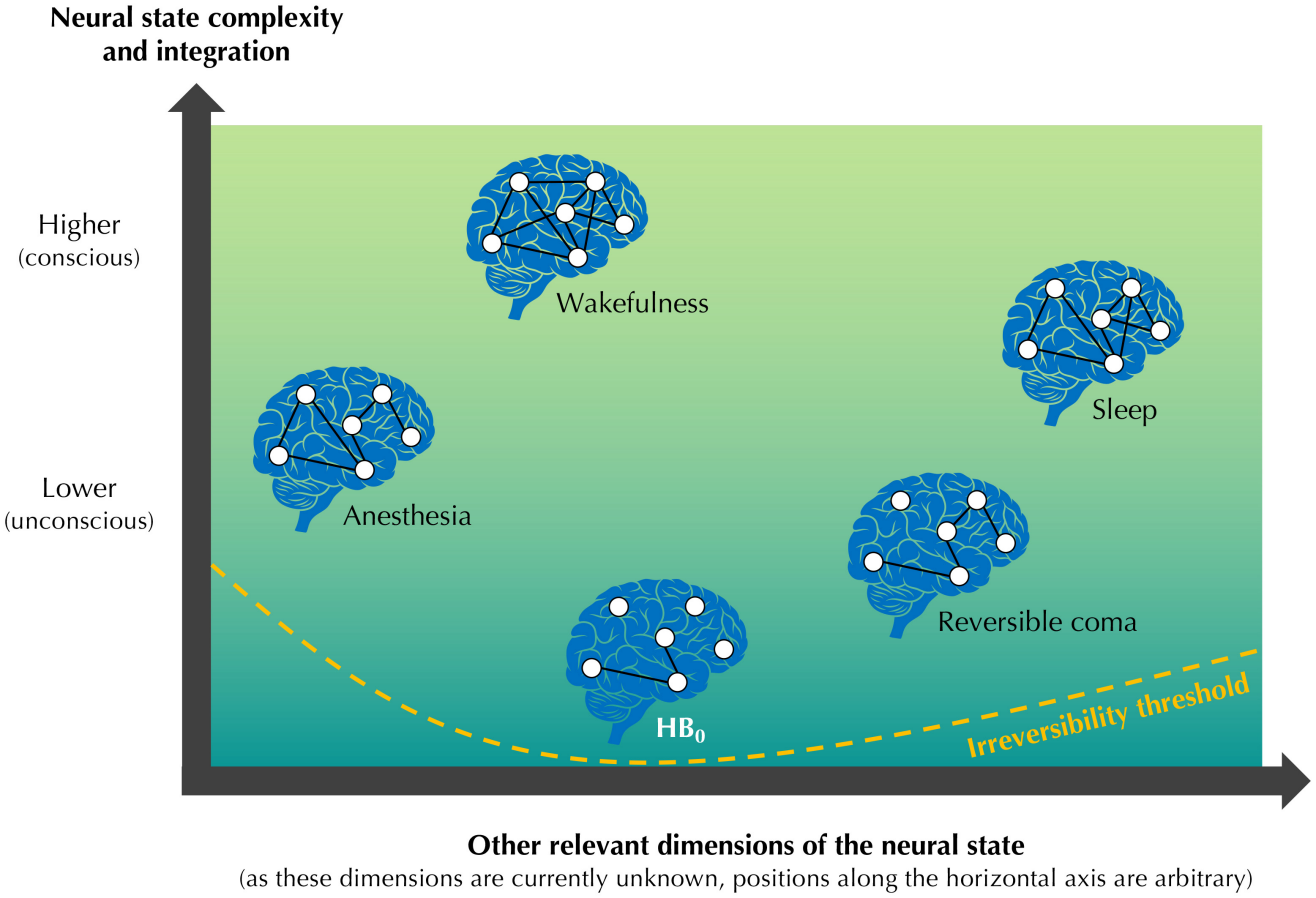
Schematic illustration of human brain zero (HB_0_). Five generic neural states are represented on a graph, approximately ordered by their level of consciousness: wakefulness, sleep, anesthesia, reversible coma, and HB_0_. The *vertical axis* represents the degree of complexity and integration of the neural state, from lower, presumably unconscious neural states (bottom) to higher, primarily conscious neural states (top): within each blue human brain symbol, white interconnected dots symbolize the functional network. The relation between the degree of complexity or integration and the degree of consciousness is a general trend suggested by available research, but not a systematic principle. The *horizontal axis* represents the projection of the other relevant dimensions of the neural state in the multidimensional space of human brain variables: the five generic neural states are displayed at arbitrary positions, to illustrate the fact that these relevant dimensions are currently unknown. At the bottom of the graph, the orange dashed curve represents the irreversibility threshold, a hypothetical boundary defined across the entire space of neural states, under which the complete recovery of consciousness is no longer possible. HB_0_ is defined as the lowest neural state, in terms of complexity and integration, that still stands above this irreversibility threshold. The figure does not distinguish between related neural states (e.g., different stages of sleep, or different forms of reversible coma), and the axes are displayed without units. The position of the neural states along the axes, and the topology of their functional networks, are only for illustrative purposes and are not intended to be biologically realistic.

Uncovering HB_0_ would require extrapolating from these trajectories and identifying, within the multidimensional space of human brain variables, the neural state where the degree of complexity and integration is the lowest, but where a complete recovery of consciousness remains possible, as illustrated in Figure 1. The search for HB_0_ candidates is likely to raise many interesting scientific questions about human consciousness. Does HB_0_ correspond to a natural state that could be discovered, or to an artificial state that should be predicted? If HB_0_ is a predicted state, how could it be engineered? Is HB_0_ relatively invariant, or are there significant differences between human brains? Is there a clear threshold separating HB_0_ from irreversible collapse, or a more progressive boundary? Is the trajectory toward HB_0_ primarily defined by brain regions, neuronal types, or other levels of organization? Does the return trajectory from HB_0_ to consciousness follow a symmetrical path, or a significantly distinct route? What are the main differences between HB_0_ and the lowest neural state of other species? Assuming that reactivity, hemodynamic activity, and electrical activity are indeed relevant proxies for evaluating the degree of complexity and integration of a given neural state, available research seems to suggest a general trend: lower complexity or integration appears to be associated with a more limited conscious experience. Considering this trend, it is reasonable to expect HB_0_ to be an unconscious state, but at a fundamental level, this is not necessarily the case. The continuum of neural states does not follow an established, systematic principle associating the degree of complexity or integration with the degree of consciousness: this trend is only a general orientation, suggested in particular by studies on wakefulness, sleep, and anesthesia. Intriguingly, we cannot rule out the possibility that HB_0_ could correspond to a conscious state relying on particularly simple and local brain activity. Therefore, a relatively unexpected scientific question could also be: does HB_0_ correspond to a conscious or an unconscious neural state?

Since the answers to such questions would likely involve the exploration of a large space of possibilities, and require the extraction of complex patterns from a diverse corpus of neuroimaging datasets, the search for HB_0_ candidates may particularly benefit from the capabilities of large foundation models dedicated to human brain data, often known as brain foundation models. Such models have demonstrated their ability to predict relevant patterns from EEG (Cui et al., 2024), fMRI (Caro et al., 2024), or MEG data (Défossez et al., 2023) in humans, or from neural activity in other animals such as mice (Wang et al., 2025). Very recently, a time-frequency foundation model, trained in particular on EEG data, has even been developed specifically for human sleep (Huang et al., 2026), further confirming the potential of such models for decoding neural state patterns, and possibly predicting consciousness transitions. Specifically, if brain foundation models are trained to classify neural states, they could be leveraged to identify the lowest states, in terms of complexity and integration, that are still classified, for example, as wakefulness, sleep, or anesthesia. In other words, by extracting relevant patterns from their training data, these models could be used to infer the lower bound of multiple physiological conditions, allowing us to gain a better understanding of the entire space of neural states. Furthermore, if brain foundation models are trained to predict neural state transitions, for example the transitions between wakefulness and sleep, they could be leveraged to identify the most essential processes underlying each of these transitions, and the conditions under which these transitions are likely to occur. These classification and prediction capabilities are realistic, since physiological conditions and transitions can both be added as labels to neuroimaging datasets, and used for the training of brain foundation models. Remarkably, the combination of these capabilities could open new horizons for consciousness engineering, as it would allow for the simulation of plausible pathways within and across various neural states, at different degrees of complexity and integration. In this sense, brain foundation models could serve as a kind of cartographic instrument for uncovering possible paths from wakefulness to HB_0_ candidates. As the capabilities of brain foundation models are constrained by the extent of their training corpus, these models would not be sufficient for fundamentally identifying HB_0_, but they could contribute to the empirical search for HB_0_ candidates by revealing the lowest neural states that can be inferred from the training data, and the trajectories that could plausibly lead to these neural states.

## 4 Future directions

Depending on the exact nature of HB_0_, the consciousness engineering problem could take different turns. As the future technologies that would be necessary to implement substrate transition are unknown and hypothetical, any discussion about the potential impact of HB_0_ on such technologies is necessarily speculative. Importantly, HB_0_ is only intended to serve as a heuristic construct for future research: its relevance to consciousness engineering is not directly established by the neuroscience literature discussed in this paper, and will need to be evaluated based on the specific requirements of substrate transition. However, based on common patterns encountered in technology development, we can already imagine some scenarios where this engineering problem could depend on the lowest degree of complexity and integration attainable. For example, if most brain regions or neural processes can be momentarily disengaged, interventions may focus on the subset of regions or processes whose activity should absolutely be preserved; conversely, if the relative continuity of the stream of experience depends on a minimally active functional network distributed throughout the human brain, interventions may become more challenging, requiring a whole-brain approach. If a clear threshold separates HB_0_ from irreversible collapse, the engineering problem may focus on keeping the trajectory above this frontier; by contrast, if the boundary is more progressive, the engineering problem may require a continuous monitoring of the neural state and a careful evaluation of the risks. If HB_0_ is relatively invariant, standardized protocols are conceivable, whereas significant differences between human brains may require personalized calibration. Overall, by providing a lower bound for the consciousness engineering problem, uncovering HB_0_ could determine which classes of future technologies are the most realistic candidates for implementing a successful substrate transition. Importantly, the usefulness of HB_0_ is not limited to the hypothetical scenario of direct whole-brain replacement, where the neural processes of an entire human brain would be transferred to a different substrate in one single step. Rather, it could also be valuable if elements of a human brain were rebuilt gradually, in a succession of controlled steps, as a low degree of complexity and integration could help ensure that none of these interventions disrupts the potential for a complete recovery of consciousness.

Since brain foundation models have the potential to emerge as important scientific instruments in the search for HB_0_ candidates, a practical recommendation for consciousness engineering could be to experiment with the capabilities of such models, and if possible, to acquire new neuroimaging datasets focusing on neural state transitions. Beyond that, the single most important methodological choice in the search for HB_0_ candidates would likely be the selection of the neuroimaging metrics that could serve as proxies for complexity and integration. The three proxies we suggested are grounded in existing neuroscience literature on wakefulness, sleep, anesthesia, and other neural states, but this selection would likely need to be adapted according to the future theoretical models of complexity and integration in the human brain, and the future requirements of consciousness engineering. Although this paper is not primarily focused on ethical and social questions, the strategy consisting in uncovering HB_0_ should, in any case, be pursued responsibly. In *Memoirs of Hadrian*, novelist Marguerite Yourcenar wrote: “*Let us try, if we can, to enter into death with open eyes*.” A similar vigilance would be needed if we decided to uncover the lowest neural state for consciousness engineering, at the unknown frontier of the stream of experience. Let us try, if we can, to enter into the deepest sleep with open eyes.
